# 4033 Evaluating the Effect of Prebiotics on the Gut Microbiome Profile and Beta-cell Function in Newly-Diagnosed Type 1 Diabetes

**DOI:** 10.1017/cts.2020.126

**Published:** 2020-07-29

**Authors:** Heba M Ismail, Carmella Evans-Molina, Linda DiMeglio

**Affiliations:** 1 Indiana University School of Medicine; 2 Indiana University

## Abstract

OBJECTIVES/GOALS: Type 1 diabetes (T1D) results from the autoimmune destruction of insulin-producing β-cells. Emerging data suggest that differences in intestinal microbiota might be critically involved both in autoimmunity and in glucose homeostasis. The prebiotic high amylose maize starch (HAMS) alters the gut microbiome profile and metabolites positively by increasing production of beneficial short chain fatty acids (SCFAs) that have significant anti-inflammatory effects. HAMS also improves glycemia, insulin sensitivity and secretion in healthy non-diabetic adults. Further, an acetylated and butyrylated form of HAMS (HAMS-AB) that increases beneficial SCFA production, namely acetate and butyrate, has been safe and effective in disease prevention in mouse T1D models. The objective of the proposed study is to assess the effect of administering a prebiotic, such as HAMS-AB, on the gut microbiome profile, SCFA production, glycemia and β-cell function in humans with T1D. METHODS/STUDY POPULATION: We hypothesize that administration of HAMS-AB will (i) improve the gut microbiome profile in humans with T1D, (ii) increase SCFA production, and (iii) improve β-cell health, β-cell function and overall glycemia. We propose a pilot randomized controlled cross-over trial of HAMS-AB in 12 youth with newly-diagnosed T1D. We will use state-of-the-art markers to profile the gut microbiome (using 16S rRNA sequencing), measure stool SCFA levels (using gas chromatography), asses β-cell stress/death (by measuring proinsulin to C-peptide ratios) and glycemia (assessed by continuous glucose monitoring and HbA1c measurements). RESULTS/ANTICIPATED RESULTS: We expect that the use of HAMS-AB in newly diagnosed youth with type 1 diabetes will alter the gut microbiome profile (thus increasing the number of fermenters and SCFA levels), β-cell function and glycemia in humans with T1D. DISCUSSION/SIGNIFICANCE OF IMPACT: Given the unknown long-term effects of immune-modulatory therapy on those at risk for or those diagnosed with T1D, the use of a prebiotic such as HAMS-AB offers a simple, safe, yet inexpensive and tolerated dietary alternative approach to mitigating disease.

